# Complete monotonicity involving some ratios of gamma functions

**DOI:** 10.1186/s13660-017-1527-4

**Published:** 2017-10-11

**Authors:** Zhen-Hang Yang, Shen-Zhou Zheng

**Affiliations:** 1 Department of Mathematics, Beijing Jiaotong University, Beijing, China; 2 Department of Science and Technology, State Grid Zhejiang Electric Power Company Research Institute, Zhejiang, Hangzhou 310014 China

**Keywords:** 33B15, 33B10, 26A48, completely monotonic function, logarithmically completely monotonic function, gamma function, psi function

## Abstract

In this paper, by using the properties of an auxiliary function, we mainly present the necessary and sufficient conditions for various ratios constructed by gamma functions to be respectively completely and logarithmically completely monotonic. As consequences, these not only unify and improve certain known results including Qi’s and Ismail’s conclusions, but also can generate some new inequalities.

## Introduction

It is well known that the classical Euler gamma function Γ is defined by

1.1 $$ \Gamma ( x ) = \int_{0}^{\infty }t^{x-1}e^{-t}\,dt $$

for $x>0$, and its logarithmic derivative $\psi ( x ) = \Gamma^{\prime } ( x ) /\Gamma ( x ) $ is known as the psi or digamma function, while $\psi^{\prime }$, $\psi^{\prime \prime }$, …are called polygamma functions.

Over the past decades, various bounds concerning certain ratios of gamma functions have been researched by many mathematicians. As a possible origin, Wendel [1] showed that, for $s\in ( 0,1 ) $ and $x>0$, the following double inequalities hold:

$$ x^{1-s}\leq \frac{\Gamma ( x+1 ) }{\Gamma ( x+s ) }\leq ( x+s ) ^{1-s}. $$

Based on a different motivation from Wendel [1], Gautschi [2] in 1959 independently got the two double inequalities: for $n\in \mathbb{N}$ and $0\leq s\leq 1$, one has

$$\begin{aligned}& e^{ ( s-1 ) \psi ( n+1 ) } < \frac{\Gamma ( n+s ) }{\Gamma ( n+1 ) }< n^{s-1},\qquad \biggl( \frac{1}{n+1} \biggr) ^{1-s} < \frac{\Gamma ( n+s ) }{ \Gamma ( n+1 ) }< \biggl( \frac{1}{n} \biggr) ^{1-s}. \end{aligned}$$

In 1983, the above inequalities were improved in [3] to be as follows: for $0< s<1$ and $x>0$,

$$ \begin{aligned} &\exp \bigl[ ( 1-s ) \psi ( x+\sqrt{s} ) \bigr] < \frac{\Gamma ( x+1 ) }{\Gamma ( x+s ) }< \exp \biggl[ ( 1-s ) \psi \biggl( x+\frac{s+1}{2} \biggr) \biggr] , \\ &\biggl( x+\frac{s}{2} \biggr) ^{1-s} < \frac{\Gamma ( x+1 ) }{ \Gamma ( x+s ) }< \biggl( x-\frac{1}{2}+ \sqrt{s+\frac{1}{4}} \biggr) ^{1-s}. \end{aligned} $$

More inequalities involving the ratios of above two gamma functions can be found in Qi’s review article [4] and the references therein. Indeed, these inequalities are almost derived by way of following the monotonicity or convexity properties of the ratios of gamma functions. Ismail et al. [5, 6] further realized that these inequalities are also the consequences of complete monotonicity of such gamma functions’ ratios.

Now let us recall that a function *f* is called completely monotonic (for short, CM) on an interval *I* if *f* has the derivative of any order on *I* and satisfies

$$ ( -1 ) ^{k} \bigl( f ( x ) \bigr) ^{ ( k ) }\geq 0 $$

for all $k\geq 0$ on *I*, see [7, 8]. An important criterion from the definition is so-called famous Bernstein’s theorem, which stated that a necessary and sufficient condition for $f(x)$ to be a completely monotonic for $0< x<\infty $ is that for nondecreasing $\alpha ( t ) $,

$$ f ( x ) = \int_{0}^{\infty }e^{-xt}\,d\alpha ( t ) $$

converges for $0< x<\infty $; for details, see [8, p.161, Theorem 12b].

Similarly, a positive function *f* is called logarithmically completely monotonic (for short, LCM) on an interval *I* if *f* has the derivative of each order on *I* and its logarithm ln*f* satisfies $( -1 ) ^{k} ( \ln f ( x ) ) ^{ ( k ) } \geq 0 $ for all $k\in \mathbb{N}$ on *I*, see [9–11]. For simplicity, in the context we denote the sets of completely and logarithmically completely monotonic functions on *I* by $\mathcal{C} [ I ] $ and $\mathcal{L} [ I ] $, respectively.

### Remark 1.1

([11])

Let *f* be a positive function on *I*. Then it is clear that $f\in \mathcal{L} [ I ] $ if and only if $- ( \ln f ) ^{\prime }\in \mathcal{C} [ I ] $.

As a classical result, Ismail et al. [5] in 1986 showed that the function

$$ x\mapsto x^{b-a}\frac{\Gamma ( x+a ) }{\Gamma ( x+b ) } $$

for any $a>b\geq 0$ is logarithmically completely monotonic on $( 0, \infty ) $ if and only if $a+b\geq 1$. Meanwhile, Bustoz and Ismail [6] further presented other complete monotonicity results involving the ratio of two gamma functions as follows.

### Theorem A

([6, Theorem 3])

*The function*

$$ x\mapsto H_{a,b,c} ( x ) = ( x+c ) ^{b-a}\frac{ \Gamma ( x+b ) }{\Gamma ( x+a ) } $$

*for* $1\geq b-a>0$ *is logarithmically completely monotonic on* $( -\min ( a,c ) ,\infty ) $ *if* $c \leq ( a+b-1 ) /2$, *and so is* $1/H_{a,b,c}$ *on* $( -\min ( b,c ) ,\infty ) $ *if* $c \geq a$.

### Theorem B

([6, Theorem 7])

*The function*

$$ x\mapsto \frac{\Gamma ( x+s ) }{\Gamma ( x+1 ) } \exp \biggl[ ( 1-s ) \psi \biggl( x+ \frac{s+1}{2} \biggr) \biggr] $$

*for* $0< s<1$ *is logarithmically completely monotonic on* $( 0,\infty ) $.

### Theorem C

([6, Theorem 6])

*The function*

1.2 $$ x\mapsto \frac{\Gamma ( x ) \Gamma ( x+a+b ) }{ \Gamma ( x+a ) \Gamma ( x+b ) } $$

*for any* $a,b\geq 0$ *is logarithmically completely monotonic on* $( 0,\infty ) $.

More complete monotonicity results concerning the combinations of gamma functions can be found in [12–15].

### Remark 1.2

Function (1.2) can be regarded to be a generalization of Gurland’s ratio defined by

1.3 $$ T ( x,y ) =\frac{\Gamma ( x ) \Gamma ( y ) }{\Gamma ( ( x+y ) /2 ) ^{2}},\quad x,y>0, $$

which appeared in Gurland’s paper [16]. For more information on Gurland’s ratio, see [6, 17, 18] and the references therein.

We would like to mention that Qi et al. obtained many important achievements concerning the complete (logarithmic) monotonicity of the ratios of gamma and polygamma functions from 2004 on, such as [10, 19–22]. Other reference materials can be found in his review article [4]. Especially, it is worth mentioning that Qi et al. [15, 23] applied an efficient auxiliary function $v_{\alpha,\beta }$ defined on $( 0,\infty ) $ by

$$ v_{\alpha,\beta } ( t ) = \textstyle\begin{cases} \frac{e^{-\alpha t}-e^{-\beta t}}{1-e^{-t}}, & \mbox{if }t\neq 0, \\ \beta -\alpha, & \mbox{if }t=0 \end{cases} $$

to give an improvement of Bustoz and Ismail’s Theorem A [6, Theorem 3]. More precisely, they proved the following theorem.

### Theorem D

([20, Theorem 1], [24, Theorem 1])

*Let* *a*, *b* *and* *c* *be real numbers and* $\rho =\min ( a,b,c ) $. *Then*

(i) $H_{a,b,c} ( x ) \in \mathcal{L} [ ( -\rho,\infty ) ] $ *if and only if*
  $$\begin{aligned} ( a,b,c ) &\in \bigl\{ (b-a) (1-a-b+2c)\geq 0 \bigr\} \\ &\quad {}\cap \bigl\{ (b-a)\bigl(\vert a-b \vert -a-b+2c\bigr)\geq 0 \bigr\} \\ &\quad {}\backslash \{ a=c+1=b+1 \} \backslash \{ b=c+1=a+1 \} . \end{aligned}$$
(ii) $H_{b,a,c} ( x ) \in \mathcal{L} [ ( - \rho,\infty ) ] $ *if and only if*
  $$\begin{aligned} ( a,b,c ) &\in \bigl\{ (b-a) (1-a-b+2c)\leq 0 \bigr\} \\ &\quad {}\cap \bigl\{ (b-a) \bigl(\vert a-b \vert -a-b+2c\bigr)\leq 0 \bigr\} \\ &\quad {}\backslash \{ a=c+1=b+1 \} \backslash \{ b=c+1=a+1 \} . \end{aligned}$$

Later, Qi and Guo [22, Theorem 1] obtained a generalization of Theorem B by establishing the logarithmically complete monotonicity of the following function:

1.4 $$ m_{s,t} ( x ) =\frac{1}{\exp [ \psi ( x+\theta ( s,t ) ) ] } \biggl[ \frac{\Gamma ( x+t ) }{\Gamma ( x+s ) } \biggr] ^{1/ ( t-s ) } $$

for $x>-\min ( s,t,\theta ( s,t ) ) $ with $s\neq t$, and they derived the following.

### Theorem E

([22, Theorem 1])

*Let* *s* *and* *t* *be two real numbers with* $s\neq t$ *and* $\theta ( s,t ) $ *be a constant depending on* *s* *and* *t*. *Then the following statements are valid*:

(i) *If* $\theta ( s,t ) \leq \min ( s,t ) $, *then* $m_{s,t} ( x ) \in \mathcal{L} [ ( - \theta ( s,t ) ,\infty ) ] $.
(ii) $1/m_{s,t} ( x ) \in \mathcal{L} [ ( - \min ( s,t ) ,\infty ) ] $ *if and only if* $\theta ( s,t ) \geq ( s+t ) /2$.

Additionally, Qi’s another result involving the logarithmically complete monotonicity of the function $m_{s,t}$ can be also found in [25, Theorem 1]. An improvement of Theorem E and new proofs of Theorems D and E can be found in recent papers [26, 27].

Inspired by the above mentioned results, we aim to present the necessary and sufficient conditions or sufficient conditions for these ratios $W_{u,v}/W_{r,s}$, $W_{u,v}/\prod_{i=1}^{n}W_{r_{i},s_{i}}^{\lambda_{i}}$ and $\prod_{i=1}^{n} ( W_{u_{i},v_{i}}/W_{r_{i},s_{i}} ) $ to be logarithmically completely monotonic on $( -\min ( u,v,r,s ) ,\infty ) $, $( -\min_{1\leq i\leq n} ( u,v,r_{i},s_{i} ) ,\infty ) $ and $( -\min_{1\leq i \leq n} ( u_{i},v_{i},r_{i},s_{i} ) ,\infty ) $, respectively, where $\lambda_{i}\geq 0$ with $\sum_{i=1}^{n}\lambda _{i}=1$ and

1.5 $$ W_{u,v} ( x ) = \textstyle\begin{cases} ( \frac{\Gamma ( x+u ) }{\Gamma ( x+v ) } ) ^{1/ ( u-v ) } & \mbox{if }u\neq v, \\ \exp [ \psi ( x+u ) ] & \mbox{if }u=v \end{cases} $$

for $x>-\min ( u,v ) $.

The rest of this paper is organized as follows. In Section 2, we introduce an auxiliary function $y_{u,v}:\mathbb{R\longrightarrow R}$ defined for $u,v\in \mathbb{R}$ by

1.6 $$ y_{u,v} ( t ) = \textstyle\begin{cases} \frac{e^{-ut}-e^{-vt}}{v-u} & \mbox{if }u\neq v, \\ te^{-ut} & \mbox{if }u=v \end{cases} $$

and show its properties. These properties, especially Property 2.6, give a necessary and sufficient condition for $y_{u,v} ( t ) \leq y_{r,s} ( t ) $ to hold for all $t>0$, which is crucial to the proof of our main results. In Section 3, by using Properties 2.3 and 2.6 of $y_{u,v} ( t ) $, the necessary and sufficient conditions for $\ln ( W_{r,s}/W_{u,v} ) $ and sufficient conditions for $\ln ( W _{u,v}/\prod_{i=1}^{n}W_{r_{i},s_{i}}^{\lambda_{i}} ) $ to be completely monotonic are realized respectively, which not only improves certain known results including Qi’s and Ismail’s conclusions, but also generates some new results. In the fourth section, by means of Properties 2.3 and 2.6 of $y_{u,v} ( t ) $ and other two techniques, we further establish the necessary and sufficient conditions for the ratios

$$ \frac{\prod_{i=1}^{n}\Gamma ( x+s_{i} ) ^{\lambda_{i}}}{\Gamma ( x+s ) },\qquad \prod_{i=1}^{n} \frac{\Gamma ( x+u_{i} ) }{\Gamma ( x+r_{i} ) }, \qquad \prod_{i=1}^{n} \frac{W_{r_{i},s} ( x ) }{W_{u_{i},s} ( x ) } $$

to be logarithmically completely monotonic.

## An important auxiliary function

It is easy to check that $y_{u,v} ( t ) $ defined by (1.6) has the following two simple properties.

### Property 2.1

We have

2.1 $$\begin{aligned}& y_{u,v} ( t ) = t \int_{0}^{1}\exp \bigl( -utx-vt ( 1-x ) \bigr)\,dx, \end{aligned}$$

2.2 $$\begin{aligned}& y_{u,v} ( t ) = \textstyle\begin{cases} e^{- ( u+v ) t/2}\frac{\sinh [ ( u-v ) t/2 ] }{ ( u-v ) /2}& \textit{if }u\neq v, \\ te^{-ut}& \textit{if }u=v. \end{cases}\displaystyle \end{aligned}$$

### Property 2.2

Let $u,v,t\in \mathbb{R}$. Then $y_{u,v} ( t ) $ satisfies the following relations:

(i) $y_{u,v} ( t ) >$ (<) 0 for $t> ( <0 ) $;
(ii) $y_{u,v} ( t ) =y_{v,u} ( t ) $;
(iii) $e^{-\rho t}y_{u-\rho,v-\rho } ( t ) =y_{u,v} ( t ) $ for any $\rho \in \mathbb{R}$.

Now let us further add other two important properties of $y_{u,v} ( t ) $ which are useful to our main proof.

### Property 2.3

Let $t\in \mathbb{R}$ with $t\neq 0$. Then

(i) the function $y_{u,v} ( t ) $ is strictly completely monotonic in both *u* and *v* on $\mathbb{R}$ for $t\in ( 0, \infty ) $;
(ii) the function $( u,v ) \mapsto \vert y_{u,v} ( t ) \vert $ is log-convex on $\mathbb{R}^{2}$ for $t\in \mathbb{R} $.

### Proof

(i) Using integral representation (2.1), we immediately get

$$ ( -1 ) ^{n}\frac{\partial^{n}y_{u,v}}{\partial u^{n}}=t^{n+1} \int_{0}^{1}x^{n}\exp \bigl( -utx-vt ( 1-x ) \bigr)\,dx>0 \quad \forall t\in ( 0,\infty ), $$

which implies the complete monotonicity of $y_{u,v} ( t ) $ with respect to *u*. By the symmetry of *u* and *v*, the function $y_{u,v} ( t ) $ has the same complete monotonicity in parameter *v*.

(ii) Let $\phi ( x ) =-utx-vt ( 1-x ) $. By integral representation (2.1), $\ln \vert y_{u,v} ( t ) \vert $ can be expressed as

$$ \ln \bigl\vert y_{u,v} ( t ) \bigr\vert =\ln \vert t\vert + \ln \int_{0}^{1}e^{\phi ( x ) }\,dx. $$

Differentiation yields

$$\begin{aligned}& \frac{\partial \ln \vert y_{u,v} ( t ) \vert }{ \partial u} = -t\frac{\int_{0}^{1}xe^{\phi ( x ) }\,dx}{\int _{0}^{1}e^{\phi ( x ) }\,dx}, \\& \frac{\partial^{2}\ln \vert y_{u,v} ( t ) \vert }{ \partial u^{2}} = t^{2}\frac{\int_{0}^{1}x^{2}e^{\phi ( x ) }\,dx\int_{0}^{1}e^{\phi ( x ) }\,dx- ( \int_{0}^{1}xe^{ \phi ( x ) }\,dx ) ^{2}}{ ( \int_{0}^{1}e^{\phi ( x ) }\,dx ) ^{2}} \\& \hphantom{\frac{\partial^{2}\ln \vert y_{u,v} ( t ) \vert }{ \partial u^{2}} }= \frac{t^{2}}{2}\frac{\int_{0}^{1}\int_{0}^{1} ( x-y ) ^{2}e^{\phi (x)+\phi (y)}\,dx\,dy}{ ( \int_{0}^{1}e^{\phi ( x ) }\,dx ) ^{2}}>0. \end{aligned}$$

Likewise, we have

$$\begin{aligned}& \frac{\partial^{2}\ln \vert y_{u,v} ( t ) \vert }{ \partial v^{2}} = \frac{\partial^{2}\ln \vert y_{u,v} ( t ) \vert }{\partial u^{2}}, \\& \frac{\partial^{2}\ln \vert y_{u,v} ( t ) \vert }{ \partial u\partial v} = -\frac{\partial^{2}\ln \vert y_{u,v} ( t ) \vert }{\partial u^{2}}. \end{aligned}$$

These indicate that

$$ \frac{\partial^{2}\ln \vert y_{u,v} ( t ) \vert }{ \partial u^{2}}\frac{\partial^{2}\ln \vert y_{u,v} ( t ) \vert }{\partial v^{2}}- \biggl[ \frac{\partial^{2}\ln \vert y _{u,v} ( t ) \vert }{\partial u\partial v} \biggr] ^{2}=0, $$

which yields the log-convexity of $\vert y_{u,v} ( t ) \vert $ in $( u,v ) $ on $\mathbb{R}^{2}$ for $t\in \mathbb{R}$ with $t\neq 0$. □

### Remark 2.4

Property 2.3 together with part (i) of Property 2.2 implies that the function $( u,v ) \mapsto y_{u,v} ( t ) $ is convex on $\mathbb{R}^{2}$ for any $t>0$.

The next result, Property 2.6, plays an essential role in proving our main theorems. To prove it, we need the following lemma.

### Lemma 2.5

([26, Theorem 2.1])

*Let* $p,q\in \mathbb{R}$ *and* $H_{p,q}$ *be defined on* $( 0,\infty ) $ *by*

2.3 $$ H_{p,q} ( t ) = \textstyle\begin{cases} ( \frac{q}{p}\frac{\sinh ( pt ) }{\sinh ( qt ) } ) ^{1/ ( p-q ) } & \textit{if }pq ( p-q ) \neq 0, \\ ( \frac{\sinh ( pt ) }{pt} ) ^{1/p} & \textit{if }p \neq 0,q=0, \\ ( \frac{\sinh ( qt ) }{qt} ) ^{1/q} & \textit{if }p=0,q \neq 0, \\ e^{t\coth ( pt ) -1/p} & \textit{if }p=q,pq\neq 0, \\ 1 & \textit{if }p=q=0. \end{cases} $$

*Then the function* $t\mapsto t^{-1}\ln H_{p,q} ( t ) $ *is strictly increasing* (*decreasing*) *from* $( 0,\infty ) $ *onto* $( 0, ( p+q ) / ( \vert p \vert +\vert q \vert ) ) $ ($( ( p+q ) / ( \vert p \vert +\vert q \vert ) ,0 ) $), *and concave* (*convex*) *on* $( 0,\infty ) $ *if* $p+q>$ (<) 0, *respectively*.

### Property 2.6

For $u,v,r,s\in \mathbb{R}$, let $y_{u,v}$ be defined on $( 0, \infty ) $ by (1.6). Then the comparison inequality $y_{u,v} ( t ) \geq y_{r,s} ( t ) $ holds for all $t>0$ if and only if

2.4 $$ u+v\leq r+s\quad \textit{and}\quad \min ( u,v ) \leq \min ( r,s ) . $$

### Proof

Let $p=\vert u-v\vert /2$, $q=\vert r-s\vert /2$. In the case of $( u-v ) ( r-s ) \neq 0$, we use the hyperbolic function representation (2.2) to obtain

$$\begin{aligned} h ( t ) &=\frac{1}{t}\ln \frac{y_{u,v} ( t ) }{y _{r,s} ( t ) } \\ &=\frac{r+s- ( u+v ) }{2}+ \frac{1}{t} \ln \biggl( \frac{\vert r-s\vert }{\vert u-v\vert }\frac{ \sinh \vert ( u-v ) t/2\vert }{\sinh \vert ( r-s ) t/2\vert } \biggr) \\ &=\frac{r+s- ( u+v ) }{2}+\frac{1}{t}\ln \biggl( \frac{q}{p} \frac{ \sinh ( pt ) }{\sinh ( qt ) } \biggr) \\ &=\frac{r+s- ( u+v ) }{2}+ ( p-q ) \frac{\ln H_{p,q} ( t ) }{t}, \end{aligned}$$

which is also true for $( u-v ) ( r-s ) =0$.

Since $p,q\geq 0$, by Lemma 2.5 we see that $t\mapsto t^{-1} \ln H_{p,q} ( t ) $ is strictly increasing from $( 0, \infty ) $ onto $( 0,1 ) $, which implies that $t\mapsto h ( t ) $ is increasing (decreasing) on $( 0, \infty ) $ if *p*≥ (≤) *q*. Consequently, $h ( t ) \geq 0$ for all $t>0$ if and only if $\lim_{t\rightarrow 0}h(t) \geq 0$ and $\lim_{t\rightarrow \infty }h(t) \geq 0$. A simple computation yields

$$\begin{aligned}& \lim_{t\rightarrow 0}h(t) = \frac{r+s- ( u+v ) }{2}, \\& \lim_{t\rightarrow \infty }h(t) = \frac{r+s- ( u+v ) }{2}+ ( p-q ) \\& \hphantom{\lim_{t\rightarrow \infty }h(t) }= \frac{r+s- ( u+v ) }{2}+\frac{\vert u-v\vert - \vert r-s\vert }{2} \\& \hphantom{\lim_{t\rightarrow \infty }h(t) } = \min ( r,s ) -\min ( u,v ), \end{aligned}$$

which proves the desired assertion. □

## Main results

Now we are in a position to state and prove our results.

### Theorem 3.1

*For fixed* $u,v,r,s\in \mathbb{R}$ *and* $\rho =\min ( u,v,r,s ) $, *let the function* $W_{u,v}$ *be defined on* $( -\min ( u,v ) ,\infty ) $ *by* (1.5). *Then* $\ln ( W_{r,s}/W _{u,v} ) \in \mathcal{C} [ ( -\rho,\infty ) ] $ *if and only if*

3.1 $$ u+v\leq r+s\quad \textit{and}\quad \min ( u,v ) \leq \min ( r,s ) . $$

### Proof

To prove the desired result, we first give the following integral representation:

3.2 $$ \ln \frac{W_{r,s} ( x ) }{W_{u,v} ( x ) }= \int_{0} ^{\infty } \bigl[ y_{u,v} ( t ) -y_{r,s} ( t ) \bigr] \frac{e^{-xt}}{t ( 1-e^{-t} ) }\,dt, $$

where $y_{u,v} ( t ) $ is defined by (1.6). In fact, using the integral representation of $\ln \Gamma ( x ) $ [28, p.258, (6.1.50)]

3.3 $$ \ln \Gamma ( x ) = \int_{0}^{\infty } \biggl[ ( x-1 ) e^{-t}- \frac{e^{-t}-e^{-xt}}{1-e^{-t}} \biggr] \frac{dt}{t}\quad \mbox{for }x>0, $$

we get that for $u-v\neq 0$,

$$\begin{aligned} \ln W_{u,v} ( x ) &=\frac{\ln \Gamma ( x+u ) - \ln \Gamma ( x+v ) }{u-v} \\ &=\frac{1}{u-v} \int_{0}^{\infty } \biggl[ ( x+u-1 ) e^{-t}- \frac{e ^{-t}-e^{- ( x+u ) t}}{1-e^{-t}} \biggr] \frac{dt}{t} \\ &\quad {}-\frac{1}{u-v} \int_{0}^{\infty } \biggl[ ( x+v-1 ) e^{-t}- \frac{e ^{-t}-e^{- ( x+v ) t}}{1-e^{-t}} \biggr] \frac{dt}{t} \\ &= \int_{0}^{\infty } \biggl( \frac{e^{-t}}{t}-y_{u,v} ( t ) \frac{e ^{-xt}}{t ( 1-e^{-t} ) } \biggr)\,dt, \end{aligned}$$

which is obviously valid for $u=v$, and then (3.2) follows.

The integral representation (3.2) and Property 2.2 indicate that

3.4 $$ \ln \frac{W_{r,s} ( x ) }{W_{u,v} ( x ) }= \int_{0} ^{\infty } \bigl[ y_{u-\rho,v-\rho } ( t ) -y_{r-\rho,s- \rho } ( t ) \bigr] \frac{e^{- ( x+\rho ) t}}{t ( 1-e^{-t} ) }\,dt. $$

Using Bernstein’s theorem, it yields that $\ln ( W_{r,s}/W_{u,v} ) \in \mathcal{C} [ ( -\rho,\infty ) ] $ if and only if $y_{u-\rho,v-\rho } ( t ) \geq y_{r-\rho,s-\rho } ( t ) $ for all $t>0$, which, from Property 2.6, is equivalent to

$$ u-\rho +v-\rho \leq r-\rho +s-\rho \quad \mbox{and} \quad\min ( u- \rho,v-\rho ) \leq \min ( r-\rho,s-\rho ) . $$

These imply that the inequalities (3.1) hold, which completes the proof. □

While $s=r+1$, the function $W_{u,v}/W_{r,r+1}$ is reduced to

$$ \frac{W_{u,v} ( x ) }{W_{r,r+1} ( x ) }= \textstyle\begin{cases} \frac{1}{x+r} [ \frac{\Gamma ( x+u ) }{\Gamma ( x+v ) } ] ^{1/ ( u-v ) } & \mbox{if }u\neq v, \\ \frac{1}{x+r}e^{\psi ( x+u ) } & \mbox{if }u=v. \end{cases} $$

By Theorem 3.1 we have the following corollary.

### Corollary 3.2

*Let* $u,v,r\in \mathbb{R}$ *and* $\rho =\min ( u,v,r ) $. *Then we have*

(i) $\ln ( W_{u,v}/W_{r,r+1} ) \in \mathcal{C} [ ( -\rho,\infty ) ] $ *if and only if* $r\leq \min [ ( u+v-1 ) /2,\min ( u,v ) ] $;
(ii) $\ln ( W_{r,r+1}/W_{u,v} ) \in \mathcal{C} [ ( -\rho,\infty ) ] $ *if and only if* $r\geq \max [ ( u+v-1 ) /2,\min ( u,v ) ] $.

### Remark 3.3

Yang and Chu [26] showed that $\ln ( W_{u,v}/W _{r,r+1} ) \in \mathcal{C} [ ( -\rho,\infty ) ] $ if and only if $2r\leq u+v-\max ( \vert u-v \vert ,1 ) $, while $\ln ( W_{r,r+1}/W_{u,v} ) \in \mathcal{C} [ ( - \rho,\infty ) ] $ if and only if $2r\geq u+v-\min ( \vert u-v \vert ,1 ) $. It is easy to check that

$$\begin{aligned}& \bigl\{ r\leq \min \bigl[ ( u+v-1 ) /2,\min ( u,v ) \bigr] \bigr\} = \bigl\{ 2r\leq u+v-\max \bigl( \vert u-v \vert ,1 \bigr) \bigr\} , \\& \bigl\{ r\geq \max \bigl[ ( u+v-1 ) /2,\min ( u,v ) \bigr] \bigr\} = \bigl\{ 2r\geq u+v-\min \bigl( \vert u-v \vert ,1 \bigr) \bigr\} . \end{aligned}$$

Therefore, these results are equivalent to Qi’s Theorem D.

For $s=r$, the function $W_{u,v}/W_{r,r}$ can be expressed as

$$ \frac{W_{u,v} ( x ) }{W_{r,r} ( x ) }= \textstyle\begin{cases} \frac{1}{\exp [ \psi ( x+r ) ] } [ \frac{ \Gamma ( x+u ) }{\Gamma ( x+v ) } ] ^{1/ ( u-v ) } & \mbox{if }u\neq v, \\ e^{\psi ( x+u ) -\psi ( x+r ) } & \mbox{if }u=v. \end{cases} $$

As a direct consequence of Theorem 3.1, we obtain the following corollary.

### Corollary 3.4

*Let* $u,v,r\in \mathbb{R}$ *and* $\rho =\min ( u,v,r ) $. *Then* $\ln ( W_{u,v}/W_{r,r} ) \in \mathcal{C} [ ( - \rho,\infty ) ] $ *if and only if* $r\leq \min ( u,v ) $, *while* $\ln ( W_{r,r}/W_{u,v} ) \in \mathcal{C} [ ( -\rho,\infty ) ] $ *if and only if* $r\geq ( u+v ) /2$.

### Remark 3.5

The above corollary slightly improves Qi and Guo’s Theorem E. Another proof of this corollary can be found in [26].

Putting $( u,v ) = ( a+b,a ) $ and $( r,s ) = ( b+c,c ) $ yields

$$ \frac{W_{a+b,a} ( x ) }{W_{b+c,c} ( x ) }= \biggl( \frac{ \Gamma ( x+a+b ) \Gamma ( x+c ) }{\Gamma ( x+a ) \Gamma ( x+b+c ) } \biggr) ^{1/b}. $$

An application of Theorem 3.1 gives a generalization of Bustoz and Ismail’s Theorem C (see [18, Lemma 1]).

### Corollary 3.6

*Let* $a,c\in \mathbb{R}$ *and* $b\geq 0$. *Then the function*

$$ x\mapsto \ln \frac{\Gamma ( x+a+b ) \Gamma ( x+c ) }{\Gamma ( x+a ) \Gamma ( x+b+c ) }\in \mathcal{C} \bigl[ \bigl( -\min ( a,c ) , \infty \bigr) \bigr] $$

*if and only if* $a\geq c$. *In particular*, *if* $a\geq c=0$, *then the function*

$$ x\mapsto \ln \frac{\Gamma ( x ) \Gamma ( x+a+b ) }{\Gamma ( x+a ) \Gamma ( x+b ) }\in \mathcal{C} \bigl[ ( 0,\infty ) \bigr] . $$

On the basis of Property 2.3, we can deduce the following theorem.

### Theorem 3.7

*Let* $r,s,r_{i},s_{i}\in \mathbb{R}$, $\lambda_{i}\geq 0$ ($i=1,2,\ldots,n$) *with* $\sum_{i=1}^{n}\lambda_{i}=1$, *and let the function* $W_{u,v}$ *be defined on* $( -\min ( u,v ) ,\infty ) $ *by* (1.5). *Then*

(i) *the function* $\ln ( W_{\bar{r},\bar{s}}/\prod_{i=1}^{n}W_{r _{i},s_{i}}^{\lambda_{i}} ) \in \mathcal{C} [ ( -\rho _{1},\infty ) ] $, *where* $\rho_{1}=\min_{1\leq i\leq n} ( r_{i},s_{i} ) $ *and*
  $$ ( \bar{r},\bar{s} ) = \Biggl( \sum_{i=1}^{n} \lambda_{i}r_{i}, \sum_{i=1}^{n} \lambda_{i}s_{i} \Biggr) ; $$
(ii) *the function* $\ln ( W_{r,s}/\prod_{i=1}^{n}W_{r,s_{i}}^{ \lambda_{i}} ) \in \mathcal{C} [ ( -\rho_{2},\infty ) ] $ *if and only if* $s\geq \bar{s}= \sum_{i=1}^{n}\lambda_{i}s_{i}$, *and so is* $-\ln ( W_{r,s}/\prod _{i=1}^{n}W_{r,s_{i}}^{\lambda_{i}} ) \in \mathcal{C} [ ( -\rho_{2},\infty ) ] $ *if* $s\leq \min_{1\leq i \leq n} ( s_{i} ) $, *where* $\rho_{2}=\min_{1\leq i\leq n} ( r,s,s_{i} ) $.

### Proof

By the integral representation (3.2) we have

$$\begin{aligned} \ln \biggl( \frac{W_{r,s} ( x ) }{\prod_{i=1}^{n}W_{r_{i},s_{i}} ^{\lambda_{i}} ( x ) } \biggr) =&\sum_{i=1}^{n} \biggl( \lambda _{i}\ln \frac{W_{r,s} ( x ) }{W_{r_{i},s_{i}} ( x ) } \biggr) \\ =&\sum_{i=1}^{n}\lambda_{i} \int_{0}^{\infty } \bigl[ y_{r_{i},s_{i}} ( t ) -y_{r,s} ( t ) \bigr] \frac{e^{-xt}}{t ( 1-e^{-t} ) }\,dt \\ =& \int_{0}^{\infty } \Biggl[ \sum _{i=1}^{n}\lambda_{i}y_{r_{i}-\rho,s _{i}-\rho } ( t ) -y_{r-\rho,s-\rho } ( t ) \Biggr] \frac{e^{- ( x+\rho ) t}}{t ( 1-e^{-t} ) }\,dt. \end{aligned}$$

(i) While $( r,s ) = ( \bar{r},\bar{s} ) $, $\rho =\rho_{1}$, by Remark 2.4 it is easy to see that $\sum_{i=1}^{n}\lambda_{i}y_{r_{i}-\rho_{1},s_{i}-\rho_{1}} ( t ) -y_{\bar{r}-\rho_{1},\bar{s}-\rho_{1}} ( t ) \geq 0$, which proves the desired assertion due to Bernstein’s theorem.

(ii) While $( r_{i},s_{i} ) = ( r,s_{i} ) $, $\rho =\rho_{2}$, we have

$$\begin{aligned} \ln \biggl( \frac{W_{r,s} ( x ) }{\prod_{i=1}^{n}W_{r,s_{i}} ^{\lambda_{i}} ( x ) } \biggr) =& \int_{0}^{\infty } \Biggl[ \sum _{i=1}^{n}\lambda_{i}y_{r-\rho_{2},s_{i}-\rho_{2}} ( t ) -y_{r-\rho_{2},s-\rho_{2}} ( t ) \Biggr] \frac{e^{- ( x+ \rho_{2} ) t}}{t ( 1-e^{-t} ) }\,dt \\ :=& \int_{0}^{\infty }I ( t ) \frac{e^{- ( x+\rho_{2} ) t}}{t ( 1-e^{-t} ) }\,dt. \end{aligned}$$

By Bernstein’s theorem, $\ln ( W_{r,s}/\prod_{i=1}^{n}W_{r,s_{i}} ^{\lambda_{i}} ) \in \mathcal{C} [ ( -\rho_{2},\infty ) ] $ if and only if $I ( t ) \geq 0$ for all $t>0$. The sufficiency obviously follows from the decreasing and convexity of $s\mapsto y_{r,s} ( t ) $ for $t>0$. The necessity can be deduced by the limit relation

$$ \lim_{t\rightarrow 0^{+}}\frac{I ( t ) }{t^{2}}= \lim_{t\rightarrow 0^{+}} \frac{\sum_{i=1}^{n}\lambda_{i}y_{r-\rho_{2},s _{i}-\rho_{2}} ( t ) -y_{r-\rho_{2},s-\rho_{2}} ( t ) }{t^{2}}=\frac{1}{2} \Biggl( s-\sum_{i=1}^{n} \lambda_{i}s_{i} \Biggr) \geq 0\mbox{.} $$

If $s\leq \min_{1\leq i\leq n} ( s_{i} ) $, then by the decreasing property of $s\mapsto y_{r,s} ( t ) $ we get $y_{r-\rho_{2},s_{i}-\rho_{2}} ( t ) \leq y_{r-\rho_{2},s- \rho_{2}} ( t ) $ for $1\leq i\leq n$, and then

$$ I ( t ) =\sum_{i=1}^{n} \lambda_{i}y_{r-\rho_{2},s_{i}-\rho_{2}} ( t ) -y_{r-\rho_{2},s-\rho_{2}} ( t ) \leq \sum _{i=1}^{n}\lambda_{i}y_{r-\rho_{2},s-\rho_{2}} ( t ) -y_{r- \rho_{2},s-\rho_{2}} ( t ) =0, $$

which implies that $-\ln ( W_{r,s}/\prod_{i=1}^{n}W_{r,s_{i}}^{ \lambda_{i}} ) \in \mathcal{C} [ ( -\rho_{2},\infty ) ] $ if $s\leq \min_{1\leq i\leq n} ( s_{i} ) $. This completes the proof. □

Note that

$$ \ln \frac{W_{u,v} ( x ) }{\prod_{i=1}^{n}W_{r_{i},s_{i}}^{ \lambda_{i}} ( x ) }=\ln \frac{W_{u,v} ( x ) }{W _{\bar{r},\bar{s}} ( x ) }+\ln \frac{W_{\bar{r},\bar{s}} ( x ) }{\prod_{i=1}^{n}W_{r_{i},s_{i}}^{\lambda_{i}} ( x ) }. $$

By Theorems 3.1 and 3.7, we obtain the following corollary.

### Corollary 3.8

*For fixed* $u,v,r_{i},s_{i}\in \mathbb{R}$, $i=1,2,\ldots,n$, *and* $\rho =\min_{1\leq i\leq n} ( u,v,r_{i},s_{i} ) $, *let the function* $W_{u,v}$ *be defined on* $( -\min ( u,v ) , \infty ) $ *by* (1.5). *Then*, *for* $\lambda_{i}>0$ *with* $\sum_{i=1}^{n}\lambda_{i}=1$, *the function* $\ln ( W_{u,v}/\Pi _{i=1}^{n}W_{r_{i},s_{i}}^{\lambda_{i}} ) \in \mathcal{C} [ ( -\rho,\infty ) ] $ *if*

$$ u+v\geq \sum_{i=1}^{n}\lambda_{i}r_{i}+\sum_{i=1}^{n} \lambda_{i}s_{i}\quad \textit{and}\quad \min ( u,v ) \geq \min \Biggl( \sum_{i=1}^{n}\lambda_{i}r_{i},\sum_{i=1}^{n}\lambda_{i}s_{i} \Biggr) . $$

## Further results

In [14], Grinshpan and Ismail considered the logarithmically complete monotonicity of a more general combination of gamma functions. More precisely, they proved the following theorem.

### Theorem F

([14, Lemma 2.1])

*Let* $\alpha_{k}$ *and* $\beta_{k}$ ($k=1,\ldots,n$) *be real numbers such that* $\sum_{k=1}^{n}\alpha_{k}=0$ *and* $\beta_{k}\geq 0$ *for all* *k*. *Then*

$$ u ( x ) =\prod_{k=1}^{n}\Gamma ( x+ \beta_{k} ) ^{\alpha_{k}} $$

*is logarithmically completely monotonic if and only if*

$$ v ( t ) =\sum_{k=1}^{n} \alpha_{k}t^{\beta_{k}}\geq 0 $$

*for all* $t\in (0,1]$.

In fact, our main results presented in Section 3 are essentially regarded as some special cases of the above theorem. Generally speaking, it is very hard to determine those parameters $\alpha_{k}$, $\beta_{k}$. Here we would like to adopt other techniques to determine those parameters such that certain combinations of gamma functions are logarithmically completely monotonic in two special cases.

The first case is that $\alpha_{n}=\max ( \alpha_{k} ) >0$ and $\alpha_{k}\leq 0$ for $1\leq k\leq n-1$. In this case, we have $( -\alpha_{k}/\alpha_{n} ) \geq 0$ with $\sum_{k=1}^{n-1} ( -\alpha_{k}/\alpha_{n} ) =1$. To this end, we need the following basic fact.

### Lemma 4.1

([29, p.133 and p.159])

*Let* $a=(a_{1},\ldots,a_{n})$ *and* $\lambda =(\lambda_{1},\ldots,\lambda_{n})$ *be positive* *n*-*tuples with* $\sum_{i=1}^{n}\lambda_{i}=1$. *Then the function* $p\mapsto M_{p}(a,\lambda )$ *defined by*

4.1 $$ M_{p} ( a,\lambda ) = \Biggl( \sum_{i=1}^{n} \lambda_{i}a_{i} ^{p} \Biggr) ^{1/p}\quad \textit{if }p\neq 0\quad \textit{and}\quad M_{0} ( a,\lambda ) =\prod _{i=1}^{n}a_{i}^{\lambda_{i}} $$

*is increasing on* $\mathbb{R}$ *with* $M_{-\infty } ( a,\lambda ) =\min_{1\leq i\leq n} ( a_{i} ) $ *and* $M_{\infty } ( a, \lambda ) =\max_{1\leq i\leq n} ( a_{i} ) $.

### Theorem 4.2

*Let* $s,s_{i}\in \mathbb{R}$, $\lambda_{i}>0$ *for* $i=1,2,\ldots,n$ *with* $\sum_{i=1}^{n}\lambda_{i}=1$. *Then*

4.2 $$ g_{0} ( x ) =\frac{\prod_{i=1}^{n}\Gamma ( x+s_{i} ) ^{\lambda_{i}}}{\Gamma ( x+s ) }\in \mathcal{L} \Bigl[ \Bigl( -\min _{1\leq i\leq n} ( s,s_{i} ) ,\infty \Bigr) \Bigr] $$

*if and only if* $s\geq \bar{s}=\sum_{i=1}^{n}\lambda_{i}s_{i}$; *and so is* $1/g_{0} ( x ) $ *on* $( -\min_{1\leq i\leq n} ( s,s _{i} ) ,\infty ) $ *if and only if* $s\leq \min_{1\leq i \leq n} ( s_{i} ) $.

### Proof

It suffices to prove that $- [ \ln g_{0} ( x ) ] ^{\prime }$, $[ \ln g_{0} ( x ) ] ^{\prime }\in \mathcal{C} [ ( -\min_{1\leq i\leq n} ( s,s_{i} ) ,\infty ) ] $ if and only if $s\geq \bar{s}=\sum_{i=1}^{n}\lambda_{i}s_{i}$ and $s\leq \min_{1\leq i\leq n} ( s_{i} ) $, respectively.

By the integral representation of $\psi ( x ) $ [28, p.258, (6.3.21)]

4.3 $$ \psi ( x ) = \int_{0}^{\infty } \biggl( \frac{e^{-t}}{t}- \frac{e ^{-xt}}{1-e^{-t}} \biggr)\,dt\mbox{ (}x>0\mbox{)}, $$

we have

$$\begin{aligned} - \bigl[ \ln g_{0} ( x ) \bigr] ^{\prime } =&\psi ( x+s ) -\sum _{i=1}^{n}\lambda_{i}\psi ( x+s_{i} ) = \int _{0}^{\infty } \Biggl( \sum _{i=1}^{n}\lambda_{i}e^{-s_{i}t}-e^{-st} \Biggr) \frac{e^{-xt}}{1-e^{-t}}\,dt \\ =& \int_{0}^{\infty } \Biggl( \sum _{i=1}^{n}\lambda_{i}e^{- ( s_{i}- \rho ) t}-e^{- ( s-\rho ) t} \Biggr) \frac{e^{- ( x+ \rho ) t}}{1-e^{-t}}\,dt \\ =& \int_{0}^{\infty } \bigl( M_{t} ( a,\lambda ) ^{t}-e^{- ( s-\rho ) t} \bigr) \frac{e^{- ( x+\rho ) t}}{1-e ^{-t}}\,dt, \end{aligned}$$

where $M_{t}(a,\lambda )$ is defined by (4.1) with $a_{i}=e^{- ( s_{i}-\rho ) }$ and $\rho =\min_{1\leq i\leq n} ( s,s _{i} ) $. By Bernstein’s theorem, we see that $- [ \ln g_{0} ( x ) ] ^{\prime }$ is completely monotonic on $( -\rho,\infty ) $ if and only if $M_{t}(a,\lambda )^{t}-e^{- ( s-\rho ) t}\geq 0$ for all $t>0$, which is equivalent to

$$ s-\rho \geq -\ln M_{t}(a,\lambda ) $$

for all $t>0$. Using Lemma 4.1, the necessary and sufficient condition for $- [ \ln g_{0} ( x ) ] ^{\prime }$ to be completely monotonic on $( -\rho,\infty ) $ is that

$$\begin{aligned} s-\rho \geq &\sup_{t\in ( 0,\infty ) } \bigl( -\ln M_{t} ( a, \lambda ) \bigr) =-\inf_{t\in ( 0,\infty ) } \bigl( \ln M_{t}(a, \lambda ) \bigr) \\ =&-\ln M_{0}(a,\lambda )=\sum_{i=1}^{n} \lambda_{i}s_{i}-\rho, \end{aligned}$$

which implies that $s\geq \sum_{i=1}^{n}\lambda_{i}s_{i}$.

Similarly, the necessary and sufficient condition for $[ \ln g _{0} ( x ) ] ^{\prime }$ to be completely monotonic on $( -\rho,\infty ) $ is that

$$\begin{aligned} s-\rho \leq &\inf_{t\in ( 0,\infty ) } \bigl( -\ln M_{t} ( a, \lambda ) \bigr) =-\sup_{t\in ( 0,\infty ) } \bigl( \ln M_{t}(a, \lambda ) \bigr) \\ =&-\max_{1\leq i\leq n} ( \ln a_{i} ) =\min _{1\leq i\leq n} ( s_{i} ) -\rho, \end{aligned}$$

which yields that $s\leq \min_{1\leq i\leq n} ( s_{i} ) $, and the proof is complete. □

Note that

4.4 $$ \lim_{x\rightarrow \infty }\frac{\Gamma ( x+a ) }{\Gamma ( x+b ) }x^{b-a}=1, $$

which yields that as $x\rightarrow \infty $,

$$ g_{0} ( x ) =\prod_{i=1}^{n} \biggl( \frac{\Gamma ( x+s _{i} ) }{\Gamma ( x+s ) } \biggr) ^{\lambda_{i}}=x^{ \bar{s}-s}\prod _{i=1}^{n} \biggl( \frac{\Gamma ( x+s_{i} ) }{ \Gamma ( x+s ) }x^{s-s_{i}} \biggr) ^{\lambda_{i}}\rightarrow \exp \bigl[ \operatorname{sgn} ( \bar{s}-s ) \infty \bigr] . $$

In order to ensure that $\lim_{x\rightarrow \infty } [ \ln g_{0} ( x ) ] \geq 0$, it is necessary to $\bar{s}-s\geq 0$. This relation in combination with Theorem 4.7 gives the following theorem.

### Theorem 4.3

*Let* $s,s_{i}\in \mathbb{R}$, $\lambda_{i}>0$, $i=1,2,\ldots,n$, *with* $\sum_{i=1}^{n}\lambda_{i}=1$. *Then the function*

$$ \ln g_{0} ( x ) =\ln \biggl( \frac{\prod_{i=1}^{n}\Gamma ( x+s_{i} ) ^{\lambda_{i}}}{\Gamma ( x+s ) } \biggr) \in \mathcal{C} \Bigl[ \Bigl( -\min_{1\leq i\leq n} ( s,s_{i} ) , \infty \Bigr) \Bigr] $$

*if and only if* $s=\bar{s}=\sum_{i=1}^{n}\lambda_{i}s_{i}$.

### Remark 4.4

We claim that the function $x\mapsto -\ln g_{0} ( x ) $ is not completely monotonic on $( -\min_{1\leq i\leq n} ( s,s_{i} ) ,\infty ) $ unless $s=s_{i}$ for $1\leq i\leq n$. If not, then $\lim_{x\rightarrow \infty } [ -\ln g_{0} ( x ) ] \geq 0$, which leads to $s\geq \bar{s}=\sum_{i=1} ^{n}\lambda_{i}s_{i}$. This together with $s\leq \min_{1\leq i\leq n} ( s_{i} ) $ yields $s=s_{i}$ for $1\leq i\leq n$, and so $g_{0} ( x ) \equiv 1$.

### Remark 4.5

Obviously, $g_{0} ( x ) $ is another generalization of Gurland’s ratio defined by (1.3).

The second case is that *n* is an even number and $\alpha_{2k-1}=1$, $\alpha_{2k}=-1$ for $k=1,2,\ldots,n/2$. Theorem C [6, Theorem 6] is clearly a direct result in this case, and as a generalization of Alzer’s work [13, Theorem 10] which was proved in 1997 as follows.

### Theorem G

([13, Theorem 10])

*Let* $a_{i}$ *and* $b_{i}$ *be the real numbers such that* $0 \leq a_{1}\leq a_{2}\leq \cdots \leq a_{n}$, $0 \leq b_{1}\leq b_{2}\leq \cdots \leq b_{n}$, *and* $\sum_{i=1}^{k}a_{i}\leq \sum_{i=1}^{k}b_{i}$ *for* $k=1,2,\ldots,n$. *Then the function* $\prod_{i=1}^{n} [ \Gamma ( x+a_{i} ) /\Gamma ( x+b_{i} ) ] $ *is logarithmically completely monotonic on* $( 0,\infty ) $.

To prove Theorem G, Alzer made use of the following lemma.

### Lemma 4.6

([30, p.64], [13, Lemma 2])

*Let* $a_{i}$ *and* $b_{i}$ ($i=1,2,\ldots,n$) *be real numbers such that* $a_{1}\leq a_{2}\leq \cdots \leq a_{n}$, $b_{1}\leq b_{2} \leq \cdots \leq b_{n}$ *and* $\sum_{i=1}^{k}a_{i}\leq \sum_{i=1}^{k}b_{i}$ *for* $k=1,2,\ldots,n$. *If the function* *f* *is decreasing and convex on* $\mathbb{R}$, *then*

$$ \sum_{i=1}^{n}f ( a_{i} ) \geq \sum_{i=1}^{n}f ( b_{i} ) . $$

Here we slightly improve Alzer’s Theorem G by way of Lemmas 4.1 and 4.6.

### Theorem 4.7

*Let* $u_{i},r_{i}\in \mathbb{R}$, $i=1,2,\ldots,n$, *such that* $u_{1} \leq u_{2}\leq \cdots \leq u_{n}$, $r_{1}\leq r_{2}\leq \cdots \leq r_{n}$, $\sum_{i=1}^{k}u_{i}\leq \sum_{i=1} ^{k}r_{i}$ *for* $k=2,3,\ldots,n-1$. *Then the function*

$$ g_{1} ( x ) =\prod_{i=1}^{n} \frac{\Gamma ( x+u_{i} ) }{\Gamma ( x+r_{i} ) }\in \mathcal{L} \bigl[ \bigl( -\min ( u_{1},r_{1} ) ,\infty \bigr) \bigr] $$

*if and only if* $u_{1}\leq r_{1}$ *and* $\sum_{i=1}^{n}u_{i}\leq \sum_{i=1}^{n}r_{i}$.

### Proof

It suffices to prove that $- [ \ln g_{1} ( x ) ] ^{\prime }\in \mathcal{C} [ ( - \min ( u_{1},r_{1} ) ,\infty ) ] $ if and only if $u_{1}\leq r_{1}$ and $\sum_{i=1}^{n}u_{i}\leq \sum_{i=1}^{n}r_{i}$. By the integral representation (4.3) we have

$$\begin{aligned} - \bigl[ \ln g_{1} ( x ) \bigr] ^{\prime } &= -\sum _{i=1} ^{n}\psi ( x+u_{i} ) +\sum _{i=1}^{n}\psi ( x+r_{i} ) \\ &=\sum _{i=1}^{n} \int_{0}^{\infty } \biggl( \frac{e^{- ( x+u_{i} ) t}}{1-e^{-t}}- \frac{e^{- ( x+r_{i} ) t}}{1-e^{-t}} \biggr)\,dt \\ & = \int_{0}^{\infty } \Biggl( \sum _{i=1}^{n}e^{- ( u_{i}-\rho ) t}-\sum _{i=1}^{n}e^{- ( r_{i}-\rho ) t} \Biggr) \frac{e^{- ( x+\rho ) t}}{1-e^{-t}} \,dt \\ &= n \int_{0}^{\infty } \bigl( M_{t} ( a,\lambda ) ^{t}-M_{t} ( b,\lambda ) ^{t} \bigr) \frac{e^{- ( x+\rho ) t}}{1-e ^{-t}}\,dt, \end{aligned}$$

where $M_{t}(a,\lambda )$ is defined by (4.1) with $a_{i}=e^{- ( u_{i}-\rho ) }$, $b_{i}=e^{- ( r_{i}-\rho ) }$, $\lambda_{i}=1/n$ and $\rho =\min_{1\leq i\leq n} ( u_{i},r_{i} ) =\min ( u_{1},r_{1} ) $. For the necessity, note that $- [ \ln g_{1} ( x ) ] ^{\prime }$ is completely monotonic on $( -\rho,\infty ) $, by Bernstein’s theorem the inequality

$$ M_{t} ( a,\lambda ) ^{t}-M_{t} ( b,\lambda ) ^{t} \geq 0, $$

or equivalently,

$$ M_{t} ( a,\lambda ) -M_{t} ( b,\lambda ) \geq 0 $$

holds for all $t>0$. Therefore, by Lemma 4.1 we have

$$\begin{aligned}& \lim_{t\rightarrow 0} \bigl( M_{t} ( a,\lambda ) -M_{t} ( b,\lambda ) \bigr) = \Biggl( \prod _{i=1}^{n}a_{i} \Biggr) ^{1/n}- \Biggl( \prod_{i=1}^{n}b_{i} \Biggr) ^{1/n}\geq 0, \\& \lim_{t\rightarrow \infty } \bigl( M_{t} ( a,\lambda ) -M _{t} ( b,\lambda ) \bigr) = \max_{1\leq i\leq n} ( a _{i} ) -\max_{1\leq i\leq n} ( b_{i} ) \geq 0, \end{aligned}$$

which are equivalent to

$$ \sum_{i=1}^{n}u_{i}\leq \sum _{i=1}^{n}r_{i}\quad \mbox{and}\quad u_{1}\leq r _{1}. $$

Since $u\mapsto e^{-ut}$ is strictly decreasing and convex on $\mathbb{R}$ for $t>0$, using Lemma 4.6 and Bernstein’s theorem, the sufficiency follows. This completes the proof. □

If $u_{i}\leq r_{i}$ ($i=1,2,\ldots,n$), then $\sum_{i=1}^{k}u_{i}\leq \sum_{i=1}^{k}r_{i}$ for $k=1,2,\ldots,n$. By Theorem 4.7 we get the following corollary.

### Corollary 4.8

*Let* $u_{i},r_{i}\in \mathbb{R}$ *such that* $u_{i}\leq r_{i}$ *for* $i=1,2,\ldots,n$. *Then the function*

$$ g_{1} ( x ) =\prod_{i=1}^{n} \frac{\Gamma ( x+u_{i} ) }{\Gamma ( x+r_{i} ) }\in \mathcal{L} \Bigl[ \Bigl( - \min_{1\leq i\leq n} ( u_{i} ) ,\infty \Bigr) \Bigr] . $$

Analogously, by virtue of (4.4) we get

$$\begin{aligned} \ln g_{1} ( x ) &=\sum_{i=1}^{n} \ln \biggl( \frac{\Gamma ( x+u_{i} ) }{\Gamma ( x+r_{i} ) } \biggr) \\ &=\sum_{i=1}^{n} \ln \biggl( \frac{\Gamma ( x+u_{i} ) }{\Gamma ( x+r_{i} ) }x^{r_{i}-u_{i}} \biggr) + \Biggl( \sum _{i=1}^{n}u _{i}-\sum _{i=1}^{n}r_{i} \Biggr) \ln x \\ &\rightarrow \operatorname{sgn} \Biggl( \sum_{i=1}^{n}u_{i}- \sum_{i=1}^{n}r _{i} \Biggr)\quad \mbox{as }x\rightarrow \infty . \end{aligned}$$

Hence, if $\lim_{x\rightarrow \infty }\ln g_{1} ( x ) \geq 0$, then there must be $\sum_{i=1}^{n}u_{i}-\sum_{i=1}^{n}r_{i}\geq 0$. This together with Theorem 4.7 yields the following theorem.

### Theorem 4.9

*Let* $u_{i},r_{i}\in \mathbb{R}$, $i=1,2,\ldots,n$, *such that* $u_{1} \leq u_{2}\leq \cdots \leq u_{n}$, $r_{1}\leq r_{2}\leq \cdots \leq r_{n}$, $\sum_{i=1}^{k}u_{i}\leq \sum_{i=1} ^{k}r_{i}$ *for* $k=2,3,\ldots,n-1$. *Then the function*

$$ \ln g_{1} ( x ) =\ln \Biggl( \prod_{i=1}^{n} \frac{\Gamma ( x+u_{i} ) }{\Gamma ( x+r_{i} ) } \Biggr) \in \mathcal{C} \bigl[ \bigl( -\min ( u_{1},r_{1} ) ,\infty \bigr) \bigr] $$

*if and only if* $u_{1}\leq r_{1}$ *and* $\sum_{i=1}^{n}u_{i}=\sum_{i=1} ^{n}r_{i} $.

Taking $n=2$ in the above two theorems, we conclude the following results.

### Corollary 4.10

*Let* $u,v,r,s\in \mathbb{R}$.

(i) *The function*
  $$ x\mapsto \frac{\Gamma ( x+u ) \Gamma ( x+v ) }{ \Gamma ( x+r ) \Gamma ( x+s ) }\in \mathcal{L} \bigl[ \bigl( -\min ( u,v,r,s ) , \infty \bigr) \bigr] $$
  *if and only if*
  $$ \min ( u,v ) \leq \min ( r,s ) \quad\textit{and}\quad u+v \leq r+s. $$
(ii) *The function*
  $$ x\mapsto \ln \frac{\Gamma ( x+u ) \Gamma ( x+v ) }{ \Gamma ( x+r ) \Gamma ( x+s ) }\in \mathcal{C} \bigl[ \bigl( -\min ( u,v,r,s ) , \infty \bigr) \bigr] $$
  *if and only if*
  $$ \min ( u,v ) \leq \min ( r,s ) \quad\textit{and}\quad u+v=r+s. $$

### Remark 4.11

Taking $( u,v ) = ( a+b,c ) $, $( r,s ) = ( a,b+c ) $ in the above corollary, we obtain Corollary 3.6.

Finally, we give the following theorem by employing the decreasing and convex properties of $v\mapsto y_{u,v} ( t ) $ on $\mathbb{R}$ for $t>0$ and Lemma 4.6.

### Theorem 4.12

*Let the function* $W_{u,v}$ *be defined on* $( -\min ( u,v ) ,\infty ) $ *by* (1.5), *and let* $u_{i},r_{i}$, $s\in \mathbb{R}$ ($i=1,2,\ldots,n$) *such that* $u_{1}\leq u_{2}\leq \cdots \leq u_{n}$, $r_{1}\leq r_{2}\leq \cdots \leq r_{n}$ *and* $\sum_{i=1}^{k}u_{i}\leq \sum_{i=1}^{k}r_{i}$ *for* $k=1,2,\ldots,n-1$. *Then the function*

$$ g_{2} ( x ) =\ln \Biggl( \prod_{i=1}^{n} \frac{W_{r_{i},s} ( x ) }{W_{u_{i},s} ( x ) } \Biggr) \in \mathcal{C} \bigl[ ( -\rho,\infty ) \bigr] $$

*if and only if* $\sum_{i=1}^{n}u_{i}\leq \sum_{i=1}^{n}r_{i}$, *where* $\rho =\min ( u_{1},r_{1},s ) $.

### Proof

By the integral representation (3.4) we have

$$\begin{aligned} g_{2} ( x ) &=\sum_{i=1}^{n} \bigl[ \ln W_{r_{i},s} ( x ) -\ln W_{u_{i},s} ( x ) \bigr] \\ &=\sum_{i=1}^{n} \int_{0}^{\infty } \bigl[ y_{u_{i}-\rho,s-\rho } ( t ) -y_{r_{i}-\rho,s-\rho } ( t ) \bigr] \frac{e ^{- ( x+\rho ) t}}{t ( 1-e^{-t} ) }\,dt \\ &:= \int_{0} ^{\infty }J ( t ) \frac{e^{- ( x+\rho ) t}}{t ( 1-e^{-t} ) }\,dt, \end{aligned}$$

where

$$ J ( t ) =\sum_{i=1}^{n}y_{u_{i}-\rho,s-\rho } ( t ) -\sum_{i=1}^{n}y_{r_{i}-\rho,s-\rho } ( t ) . $$

By Bernstein’s theorem, if $g_{2}$ is completely monotonic on $( -\rho,\infty ) $, then $J ( t ) \geq 0$ for all $t>0$. It follows that

$$ \lim_{t\rightarrow 0}\frac{J ( t ) }{t^{2}}=\sum _{i=1}^{n} \biggl( \lim_{t\rightarrow 0} \frac{y_{u_{i}-\rho,s-\rho } ( t ) -y_{r_{i}-\rho,s-\rho } ( t ) }{t^{2}} \biggr) =\frac{1}{2} \sum_{i=1}^{n} ( u_{i}-r_{i} ) \geq 0, $$

which implies the necessity.

Based on Property 2.3, we see that $r\mapsto y_{r,s} ( t ) $ is strictly decreasing and convex on $\mathbb{R}$ for $t>0$. Applying Lemma 4.6 with $J ( t ) \geq 0$ for all $t>0$, it yields the sufficiency due to Bernstein’s theorem. This completes the proof. □

## Conclusions

In this paper, we investigate the (logarithmically) complete monotonicity of various ratios structured by gamma functions including

$$ \frac{W_{r,s} ( x ) }{W_{u,v} ( x ) },\qquad \frac{ \prod_{i=1}^{n}\Gamma ( x+s_{i} ) ^{\lambda_{i}}}{\Gamma ( x+s ) }, \qquad \prod _{i=1}^{n}\frac{\Gamma ( x+u_{i} ) }{\Gamma ( x+r _{i} ) },\qquad \prod _{i=1}^{n}\frac{W_{r_{i},s} ( x ) }{W_{u_{i},s} ( x ) }, $$

where $W_{u,v} ( x ) $ is defined by (1.5).

By using Properties 2.3 and 2.6 of $y_{u,v} ( t ) $ defined by (1.6), we present in Theorem 3.1 a nice result, which states that $\ln ( W_{r,s}/W_{u,v} ) \in \mathcal{C} [ ( -\rho,\infty ) ] $ if and only if $u+v\leq r+s$ and $\min ( u,v ) \leq \min ( r,s ) $. This unifies and improves some known results presented by Qi and Ismail, that are Theorems A-E.

Applying the increasing property of $M_{p} ( a,\lambda ) $ defined by (4.1) in its order *p*, we show in Theorem 4.3 that

$$ \ln g_{0} ( x ) =\ln \biggl( \frac{\prod_{i=1}^{n}\Gamma ( x+s_{i} ) ^{\lambda_{i}}}{\Gamma ( x+s ) } \biggr) \in \mathcal{C} \Bigl[ \Bigl( -\min_{1\leq i\leq n} ( s,s_{i} ) , \infty \Bigr) \Bigr] \quad \iff \quad s=\bar{s}=\sum_{i=1}^{n} \lambda_{i}s_{i}. $$

Clearly, $g_{0} ( x ) $ for $s=\bar{s}$ is a generalization of Gurland’s ratio defined by (1.3).

It is interesting that both $\ln ( W_{r,s}/\prod_{i=1}^{n}W_{r,s _{i}}^{\lambda_{i}} ) \in \mathcal{C} [ ( - \min_{1\leq i\leq n} ( r,s,s_{i} ) ,\infty ) ] $ and $g_{0} ( x ) \in \mathcal{L} [ ( - \min_{1\leq i\leq n} ( s,s_{i} ) ,\infty ) ] $ if and only if $s\geq \bar{s}=\sum_{i=1}^{n}\lambda_{i}s_{i}$, which are stated in Theorems 3.7 and 4.2.

Theorems 4.7 and 4.9 follow from Lemmas 4.1 and 4.6, which improve Alzer’s result, that is, Theorem G. Comparing Theorem 3.1 and Corollary 4.10, we find that both

$$ \ln \biggl( \frac{W_{r,s}}{W_{u,v}} \biggr) \in \mathcal{C} \bigl[ ( -\rho,\infty ) \bigr] \quad \mbox{and}\quad x\mapsto \frac{\Gamma ( x+u ) \Gamma ( x+v ) }{\Gamma ( x+r ) \Gamma ( x+s ) }\in \mathcal{L} \bigl[ ( -\rho, \infty ) \bigr] $$

if and only if $u+v\leq r+s$ and $\min ( u,v ) \leq \min ( r,s ) $, where $\rho =\min ( u,v,r,s ) $.
